# Use of WeChat-based patient-doctor interaction improves patient experience of *Helicobacter pylori* treatment: a randomized controlled trial

**DOI:** 10.3389/fdgth.2026.1744252

**Published:** 2026-05-29

**Authors:** Boshen Lin, Wenlin Zhang, Jinghui Zhang, Minjuan Lin, Jing Liu, Zhongxue Han, Yuming Ding, Qingzhou Kong, Miao Duan, Yueyue Li, Xiuli Zuo, Yanqing Li

**Affiliations:** 1 Department of Gastroenterology, Qilu Hospital, Cheeloo College of Medicine, Shandong University, Jinan, China; 2 Laboratory of Translational Gastroenterology, Qilu Hospital, Cheeloo College of Medicine, Shandong University, Jinan, China; 3 Robot Engineering Laboratory for Precise Diagnosis and Therapy of GI Tumor, Qilu Hospital, Cheeloo College of Medicine, Shandong University, Jinan, China

**Keywords:** helicobacter pylori, patient experience, patient-doctor interaction, telemedicine, therapy

## Abstract

**Introduction:**

*Helicobacter pylori* (*H. pylori*) eradication therapy often involves complex medication regimens and may be accompanied by adverse events, which can negatively affect treatment outcomes and experience. Effective patient-doctor communication may help address these challenges. This study primarily aimed to evaluate whether a WeChat-based patient-doctor interaction (WPDI) system could improve *H. pylori* treatment outcomes while simultaneously enhancing patient experience.

**Methods:**

In this prospective, open-label randomized controlled trial, treatment-naïve *H. pylori*-infected patients were randomly assigned (1:1) to either a WPDI group or a control group. All participants received routine patient education and a 14-day vonoprazan-containing quadruple therapy. In addition to routine education, patients in the WPDI group were invited to participate in a physician-moderated WeChat group that allowed real-time communication and consultation during treatment. Patient experience, compliance, and adverse events were assessed through a standardized telephone interview after completion of therapy, and *H. pylori* eradication was confirmed by a ^13^C-urea breath test six weeks after treatment. This study was registered at clinicaltrials.gov. (No. NCT04850209).

**Results:**

In total, 438 patients were enrolled for randomization. The WPDI group achieved eradication rates of 90.4% (198/219), 93.4% (198/212), and 94.2% (194/206), as evaluated by intention-to-treat, modified intention-to-treat, and per-protocol analysis, respectively. The eradication rates were comparable to those in the control group (89.5% [196/219], 93.3% [196/210], and 94.6% [192/203]. The assessment results of patient experience showed that more patients voted that the treatment was satisfactory or very satisfactory in the WPDI group (90.3% vs. 74.9%; *P* < 0.001), whereas more participants tended to feel worried (16.0% vs. 26.1%; *P* = 0.012) and perturbed (11.7% vs. 22.7%; *P* = 0.003) during the treatment period in the control group. The two groups exhibited similar levels of compliance and adverse events.

**Conclusions:**

The WeChat-based patient-doctor interaction improved patient experience of *H. pylori* eradication therapy significantly while the treatment outcomes were not promoted significantly.

**Clinical Trial Registration:**

ClinicalTrials.gov ID: NCT04850209.

## 1 Introduction

*Helicobacter pylori* (*H. pylori*) infection presents a global hazard as it is a common infectious disease (1). As a class I carcinogen, *H. pylori* threatens the gastrointestinal fitness of approximately 50% of the global population. Although the global infection rate of *H. pylori* is showing a downward trend, the infection rates in Asian countries remain high (2, 3). Although most patients are asymptomatic or present with atypical symptoms, *H. pylori*-positive individuals are at a higher risk of many gastrointestinal diseases and extra-digestive system disorders. Previous studies have confirmed that eradicating *H. pylori* is a controllable factor that decreases the incidence rate of gastric carcinoma (4, 5).

Treating *H. pylori* infection is an intricate process for patients; it usually entails a two-week medication regimen and the administration of multiple drugs (6, 7). Furthermore, the treatment period is possibly accompanied by adverse events (AEs) (8). Although patients face the pressure of adhering to the treatment plan and experiencing discomfort caused by side effects, they often find it challenging to receive timely assistance from physicians due to difficulties in scheduling appointments (9). This may result in confusion and anxiety during the medication process, ultimately contributing to treatment failure and a deterioration in overall treatment experience (10, 11).

Although several studies have explored strategies to enhance patient education during *H. pylori* eradication therapy, most have primarily focused on improving treatment adherence or increasing eradication rates (12, 13). Comparatively little attention has been paid to patients’ treatment experience during the eradication process. Effective patient-doctor communication has been shown to influence patients' decision-making and improve satisfaction with medical care (14–16). However, evidence from randomized controlled trials evaluating structured digital communication between patients and physicians during the treatment period remains limited. Therefore, this study was designed to prospectively evaluate whether a WeChat-based patient-doctor interaction (WPDI) system could improve treatment outcomes—including eradication rates, compliance, and adverse events—while also assessing its impact on patient experience during *H. pylori* eradication therapy.

## 2 Materials and methods

This was an open-label, prospective, randomized controlled trial performed at the Qilu Hospital of Shandong University in Jinan, Shandong province, China. The Medical Ethics Committee of Qilu Hospital of Shandong University approved the study protocol. This study was registered at clinicaltrials.gov. (No. NCT04850209). Before inclusion to the study, all participants have signed informed consent.

### 2.1 Patients and randomization

Consecutive adult patients (aged between 18 and 75 years old) who underwent initial eradication were enrolled between July 2021 and July 2022. *H. pylori-*positive results were confirmed by a positive ^13^C-urea breath test (^13^C-UBT), rapid urease test, or histopathology. The main exclusion criteria were as follows: previous history of *H. pylori* eradication therapy; history of allergy to the medications used in the therapeutic regimen; previous history of stomach surgery; pregnancy or lactation; the presence of severe underlying diseases such as renal failure and malignant tumors; usage of antibiotics, bismuth, or Chinese herbal medicine within four weeks before therapy initiation; intake of proton pump inhibitors, potassium-competitive acid blocker, or H-2 blocker drugs within two weeks before therapy initiation; unwilling or incapable of providing informed consent; and illiterate or incapable of using a smartphone.

Eligible patients were randomly assigned 1:1 to the WPDI or control group after they signed the informed consent form. A randomized sequence generated by a computer was utilized to ensure proper randomization, and subsequently, concealed in an opaque envelope. The sealed envelope was then entrusted to an independent research assistant who was not involved in the study.

### 2.2 Patient-doctor interaction

Routine patient education: All the enrolled patients received the following education: (i) Oral education: Physicians provided detailed oral explanations of the specification and usage of medicines, their possible side effects, dietary instructions, time points of reexamination and their methods, the importance of good compliance, and the harm of poor compliance. (ii) Written education: Guiding cards were offered to all patients to bring home. All the oral education content was repeatedly stressed on the guiding cards. A specialized chart was offered to instruct patients to record the number of drugs taken daily and AEs.

Patient-doctor interaction: Except for routine patient education, patients assigned to the WPDI group were invited to a group chat administrated by physicians specialized in *H. pylori.* The WeChat group was managed by a team of 11 physicians. Anticipated patient queries were pre-discussed and standardized through structured training. One physician was designated to oversee all responses to ensure full compliance with current clinical guidelines. All participants were required not to reveal personal information in the group chat for privacy concerns. However, participants were encouraged to ask questions regarding any aspect of *H. pylori* treatment at any time. They were also encouraged to post questions if they experienced any discomfort. Physicians provided solutions and answers to these problems and questions promptly.

### 2.3 Therapeutic regimens

All participants in the two groups received a vonoprazan-containing quadruple therapy comprising: (1) vonoprazan (Takeda Pharmaceutical, Tianjin, China), 20 mg b.i.d. (2) amoxicillin (The United Laboratories Co. Ltd., Zhuhai, China), 1,000 mg b.i.d.(3) clarithromycin (Abbott Pharmaceutical Co. Ltd., Shanghai, China), 500 mg b.i.d. (4) colloidal bismuth pectin (Zhendong Ante Biological Pharmaceutical Co., Ltd., Jinzhong, Shanxi Province, China), 200 mg b.i.d. or bismuth potassium citrate (Livzon Pharmaceutical Group Inc., Zhuhai, Guangdong Province, China), 220 mg b.i.d. The treatment lasted 14 days.

### 2.4 Telephone interview and reexamination

A standardized telephone interview was conducted one day after completion of the medication regimen to collect information on patient experience, compliance, and AEs. AEs were assessed according to the Common Terminology Criteria for Adverse Events (CTCAE v5.0) and graded as mild, moderate, or severe. Good compliance was defined as the consumption of at least 80% of the total dosage through pill counts. The assessing investigators were blinded to group allocation to ensure the objectivity of the results.

Patient experiences were assessed using three items: (i) Patient satisfaction score based on a 5-point Likert scale (1 = very unsatisfactory; 2 = unsatisfactory; 3 = fair; 4 = satisfactory; 5 = very satisfactory), and patients with scores of 4 or 5 were defined as satisfied with treatment; (ii) question 1: “Have you felt worried about therapy during the treatment period?”; (iii)question 2: “Have you felt perturbed about therapy during the treatment period?”. The answers to the latter two questions were either “yes” or “no.” Six weeks after the last intake of the study drugs, a reexamination based on ^13^C-UBT was conducted. 4 ‰ was set as the cut-off value of ^13^C-UBT.

### 2.5 Outcomes

The major outcome was the eradication rate of *H. pylori* in the WPI and control groups. The secondary outcomes included patient experience with eradication treatment, frequencies and categories of AEs and patient compliance.

### 2.6 Statistical analysis

According to previous studies using WeChat for patient education (17, 18), we estimated that the eradication rates of the WPDI group and control groups were 90% and 80%, respectively, with a significance level of 0.05 (*α* = 0.05). Assuming a 10% withdrawal or loss to follow-up, we calculated that at least 219 patients were required in each group to achieve 80% (1−*β*) power. The calculation of the sample size was performed through Power Analysis and Sample Size (PASS) 11 (NCSS; LLC., Kaysville, Utah, USA).

Intention-to-treat (ITT), modified intention-to-treat (MITT) and per-protocol (PP) analyses were used to evaluate the *H. pylori* eradication rate. All enrolled individuals were included in the ITT analysis. Patients who had taken at least one dose of prescribed drugs and finished the reexamination were included in the MITT analysis. Patients who failed to consume 80% of the medications were excluded from the PP analysis set. For qualitative parameters, either the *χ*^2^ test or Fisher's exact test were used, while the student's t-test was employed for quantitative parameters. *P*-values of <0.05 were considered statistically significant. Data analysis was conducted using SPSS software (version 23.0; IBM Corp., Armonk, New York, USA).

## 3 Results

### 3.1 Patients' baseline demographic and clinical characteristics

**Figure 1:**
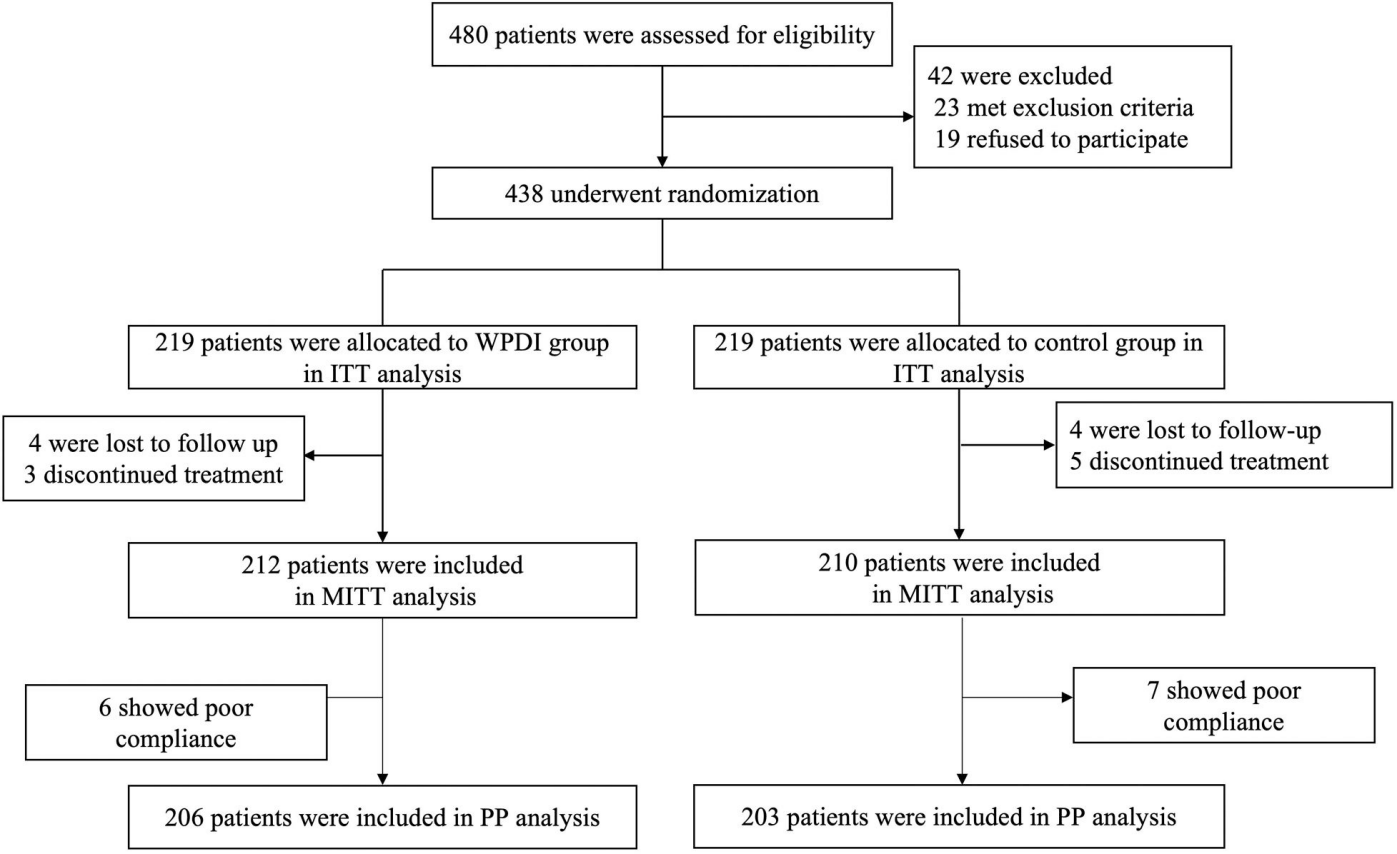
Flow diagram of patient selection and study design. WPDI, WeChat-based patient-doctor interaction; ITT, intention-to-treat; MITT, modified intention-to-treat; PP, per-protocol.

The screening and recruitment of study participants are illustrated in Figure 1. In total, 480 *H pylori*-infected outpatients were assessed for eligibility from July 2021 to July 2022; among them, 23 met the exclusion criteria, and 19 were unwilling to participate. Finally, 438 patients were randomized to the WPDI and control groups. As shown in Table 1, there were no statistically significant differences in patient demographics and clinical characteristics between the two study groups.

**Table 1:** Baseline characteristics of participants.

Characteristics	WPDI group (*N* = 219)	Control group (*N* = 219)	*P* value
Female sex, *n* (%)	115 (52.5)	104 (47.5)	0.293
Age (y)(mean ± SD)	42.66 ± 12.84	43.29 ± 13.20	0.613
BMI, kg/m^2^(mean ± SD)	23.91 ± 3.48	24.02 ± 3.37	0.734
Grade of education			
High school or less, *n* (%)	73 (33.3)	68 (31.1)	0.609
University or more, *n* (%)	146 (66.7)	151 (68.9)	0.609
Cigarette, *n* (%)	20 (9.1)	24 (11.0)	0.525
Alcohol, *n* (%)	46 (21.0)	44 (20.1)	0.813
Concomitant diseases, *n* (%)			
Diabetes mellitus, *n* (%)	7 (3.2)	9 (4.1)	0.610
Hypertension, *n* (%)	16 (7.3)	15 (6.8)	0.852
Family gastric cancer history, *n* (%)	17 (7.8)	19(8.7)	0.728

BMI, body mass index; WPDI, WeChat-based patient-doctor interaction; SD, standard deviation.

### 3.2 *H. pylori* eradication rates

**Figure 2:**
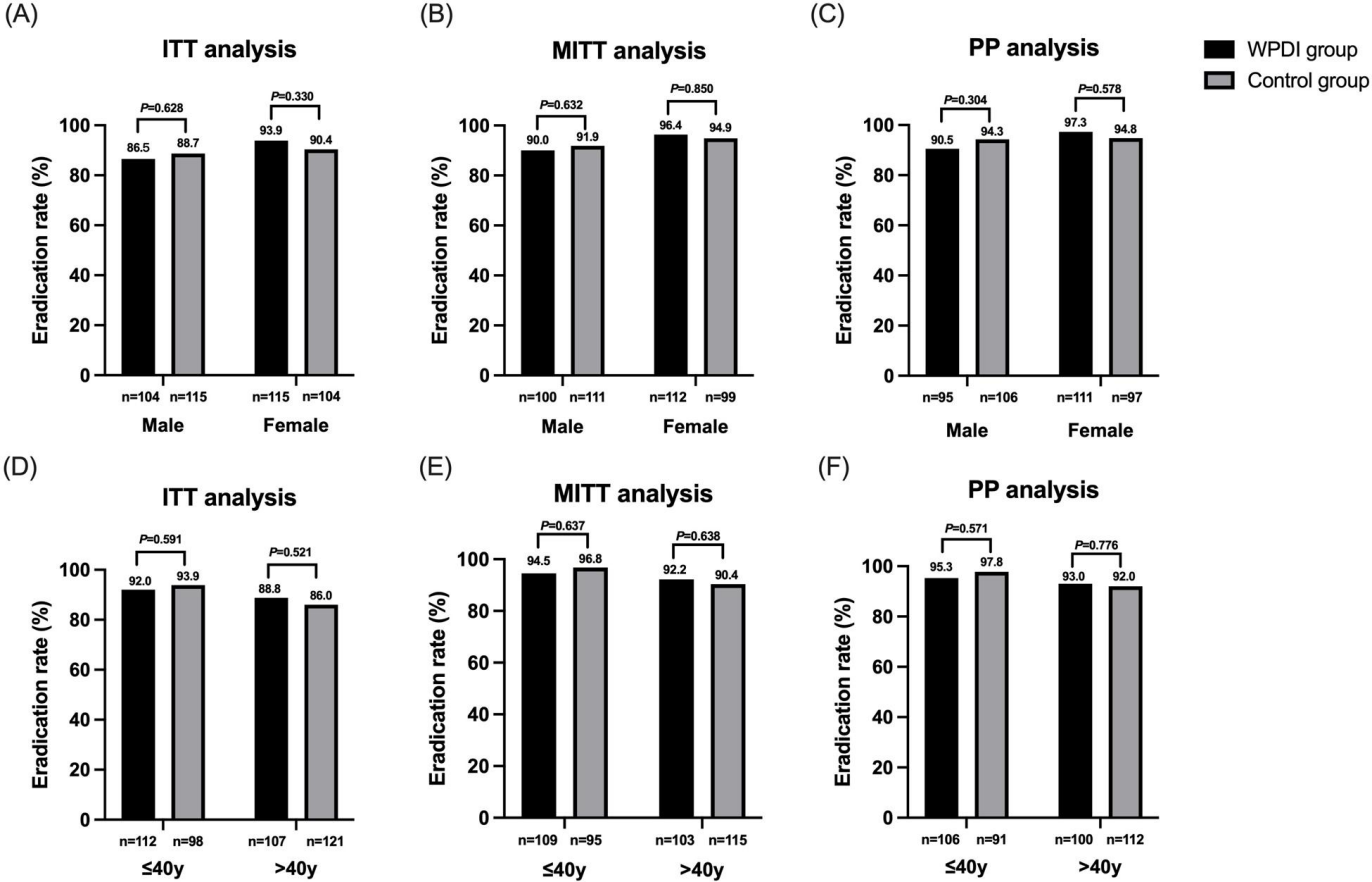
Subgroup analyses of eradication rates stratified by age and sex. **(A–C)** Eradication rates according to sex. **(D–F)** Eradication rates according to age; ITT, intention-to-treat; MITT, modified intention-to-treat; PP, per-protocol.

The *H. pylori* eradication rates in the WPDI and control groups are shown in Table 2. In the ITT analysis, the *H. pylori* eradication rates were 90.4% (198/219, 95% CI, 85.7–94.0%) in the WPDI group and 89.5% (196/219, 95% CI, 84.7–93.2%) in the control group (*P* = 0.751). The *H. pylori* eradication rates in the MITT analysis for the WPDI group and control group were 93.4% (198/212, 95% CI, 89.2–96.3%) and 93.3% (196/210, 95% CI, 89.1–96.3%), respectively (*P* = 0.979). In the PP analysis, the *H. pylori* eradication rates were 94.2% (194/206, 95% CI, 90.0–97.0%) in the WPDI group and 94.6% (192/203, 95% CI, 90.5–97.3%) in the control group (*P* = 0.858). Furthermore, as shown in Figure 2, subgroup analyses stratified by age and sex revealed no significant differences in eradication rates between the two groups.

**Table 2:** *H. pylori* eradication rates in different groups.

Eradication rate, *n* (%)	WPDI group n/N (%)	Control group n/N (%)	*P* value
ITT analysis			
Eradication rate	198/219 (90.4)	196/219 (89.5)	0.751
95% CI	85.7%–94.0%	84.7%–93.2%	
MITT analysis			
Eradication rate	198/212 (93.4)	196/210 (93.3)	0.979
95% CI	89.2%–96.3%	89.1%–96.3%	
PP analysis			
Eradication rate	194/206 (94.2)	192/203 (94.6)	0.858
95% CI	90.0%–97.0%	90.5%–97.3%	

ITT, Intention-to-treat; MITT, modified intention-to-treat; PP, Per-protocol; CI, confidence interval; WPDI, WeChat-based patient-doctor interaction.

### 3.3 Patient-doctor interaction and patient experience

After reviewing the chat history, we found that 86.3% (189/219) of participants in the WPDI group had posted questions, presenting a total of 911 questions. In addition, physicians provided a total of 1,147 suggestions during the 12 months study period. Therefore, every patient received an average of approximately five suggestions, resulting in an average of approximately three suggestions being released daily.

The proportion of satisfied patients in the WPDI group and control group was 90.3% (186/206, 95% CI, 86.2–94.4%) and 74.9% (152/203, 95% CI, 68.9–80.9%), respectively (*P* < 0.001). Furthermore, only 16.0% (33/206, 95% CI, 11.0–21.1%) of patients in the WPDI group compared to 26.1% (53/203, 95% CI, 20.0–32.2%, *P* = 0.012) of patients in the control group who felt worried about the therapy during the treatment period, whereas 11.7% (24/206, 95% CI, 7.2–16.1%) of patients in the WPDI group and 22.7% (46/203, 95% CI, 16.9–28.5%, *P* = 0.003) of patients in the control group felt perturbed about the therapy during the treatment period.

### 3.4 AEs and patient compliance

The type and incidence of AEs are summarized in Table 3 and are comparable between the WPDI and control groups (25.2% vs. 29.6%, *P* = 0.328). Overall, all AEs were graded as mild. Patient compliance also had no significant differences between the WPDI and control groups (97.2% vs. 96.7%, *P* = 0.765).

**Table 3:** Adverse events in the WPDI group and the control group.

Adverse events ^a^, *n* (%)	WPDI group (*n* = 206) *n* (%)	Control group (*n* = 203) *n* (%)	*P* value
Total	52 (25.2)	60 (29.6)	0.328
Stomachache	5 (2.4)	7 (3.4)	0.541
Abdominal distention	5 (2.4)	6 (3.0)	0.741
Diarrhea	6 (2.9)	5 (2.5)	0.779
Constipation	0 (0)	2 (1.0)	0.246
Nausea	6 (2.9)	8 (3.9)	0.567
Vomiting	1 (0.5)	4 (2.0)	0.213
Dysgeusia	30 (14.6)	34 (16.7)	0.543
Skin rash	1 (0.5)	1 (0.5)	>0.99
Pruritus	1 (0.5)	1 (0.5)	>0.99
Others	11 (5.3)	7 (3.4)	0.351

a Per-protocol analysis.

WPDI, WeChat-based patient-doctor interaction;.

## 4 Discussion

In this study, we found that WPDI significantly improved patients' treatment experience. Moreover, both the WPDI and control groups achieved a good eradication rate; however, applying WPDI in the management of *H. pylori* eradication therapy did not lead to improvements in either the eradication rate or compliance.

Good treatment experience should be an important goal of health services (19). Although there have been some advancements in the field of *H. pylori* treatment, doctors still pay insufficient attention to patients' treatment experiences. In clinical practice, we have observed that patients undergoing *H. pylori* eradication treatment often encounter a range of issues. These include uncertainties about how to cope with AEs, doubts about whether to continue medication and concerns about potential interactions with other drugs. These challenges typically do not arise at the initial patient consultation but gradually manifest during the course of treatment. Such uncertainties may lead patients to self-discontinue medication or experience anxiety and uneasiness. With the advancement of technology, telemedicine systems may contribute to addressing this issue.

Our study revealed that patients who participated in the WeChat group chat of the WPDI group had significantly better treatment experiences than those in the control group. This improvement may be attributed to patients receiving prompt responses from physicians regarding their queries about medication usage, advice on AEs, and comfort with medication. On the contrary, patients in the control group did not get any help from investigators after the initiation of the therapy. Therefore, fewer patients in the WPDI group felt worried and perturbed about the therapy during the treatment period. In addition, the lack of communication may generate dissatisfaction and lead to negative experiences (20); however, the implementation of WDPI added the communication duration between doctors and patients, which could potentially ease their pressure and ultimately improve their treatment satisfaction. As a result, most patients voted that the treatment was satisfactory or very satisfactory in the WPDI group, whereas the proportion of such patients in the control group was significantly lower.

This finding aligns with a growing body of evidence highlighting the positive impact of mHealth interventions on patient-reported outcomes across various chronic conditions. For instance, a participatory design study developing a mobile health application for diabetic retinopathy patients demonstrated high user satisfaction and perceived usefulness, suggesting that such tools have the potential to enhance patients' ability to manage their condition and improve their overall care experience by providing accessible information and support (21). Similarly, research on the development of a self-management mobile application for individuals with spinal cord injury in Persian demonstrated that the app achieved high usability scores and positive user feedback, suggesting its potential to serve as a valuable tool for enhancing patients' confidence and sense of control in managing their complex health needs, thereby contributing to a more positive healthcare experience (22). Furthermore, studies on gestational diabetes have emphasized that well-designed smartphone applications can address critical informational gaps, reducing patient anxiety and fostering a sense of empowerment throughout the treatment journey (23). These parallels suggest that the mechanism by which WPDI improved patient experience—by bridging the communication gap and providing real-time, accessible support—is a consistent benefit of mHealth interventions across diverse disease contexts.

Three previous studies have applied WeChat management in treating *H. pylori* infection. Studies conducted by Ma et al., and Luo et al., demonstrated that WeChat management could significantly improve the eradication rate, compliance, and follow-up rate (18, 24). Another study designed a WeChat-based mini-app as a reminder tool and revealed that patient compliance improved while the eradication rate did not (17). However, all three studies only provided verbal guidance to participants in the control group. Due to the variety of drugs, the complexity of regimens, and long treatment courses, providing verbal instructions alone may be inadequate for non-professional individuals to fully comprehend the treatment process. Insufficient patient education often leads to low compliance and eventually affects treatment efficacy (25, 26). In our study, we offered adequate patient education, including oral and written education for every participant, which covered medication usage, side effects, dietary instruction, and reexamination. As a result, more than 96% of participants in the control group also achieved good compliance. As compliance in both groups is high, it is unsurprising that the eradication rates were comparable between the two groups. This suggests that adequate routine education is efficient and essential in *H. pylori* eradication therapy.

Our results resonate with those of the iACT4IBD randomized controlled trial, which evaluated an online Acceptance and Commitment Therapy intervention for inflammatory bowel disease. In that study, while the intervention significantly bolstered disease-specific well-being, it yielded no significant reduction in clinical disease activity. This lack of clinical divergence was largely attributed to the high standard of conventional medical care received by both groups, which left limited room for further measurable improvement (27). This observation points toward a ceiling effect, suggesting that when the standard of care is already at a high level, the capacity for mHealth interventions to elicit further clinical changes is significantly diminished. Consequently, the utility of mHealth tools in such high-performance settings shifts from clinical enhancement to addressing the psychosocial dimensions of care. Similarly, our WPDI system functioned as a vital adjunct, bridging the emotional and informational gaps that persisted despite the provision of thorough, high-standard conventional instructions.

Of note, the generalizability of our findings should be considered in the context of the study population. In this study, most participants had relatively high educational levels, with approximately two-thirds having a university education or higher. Patients with higher educational attainment may find it easier to understand treatment instructions and engage in digital communication platforms such as WeChat (28, 29). Therefore, the benefits of the WPDI system observed in this study may not be fully generalizable to populations with lower educational levels, who may have more difficulty using smartphone-based communication tools or interpreting medical information delivered through such platforms. In addition, the study was conducted at a tertiary medical center in an urban setting, where smartphone use and internet access are widespread. In rural areas or regions with limited digital infrastructure, patients may have less access to smartphones or may be less familiar with social media applications such as WeChat. As a result, the feasibility and effectiveness of the WPDI approach in these settings may differ from those observed in our study. Future studies including populations from rural areas and with more diverse educational backgrounds are needed to further evaluate the applicability and effectiveness of this approach across different healthcare settings.

This study has several limitations. First, it was conducted at a single center, which may limit the generalizability of the findings, and therefore the results should be confirmed in larger multicenter studies. Second, the study's open-label design may have introduced reporting bias. Participants' awareness of their group allocation could lead those in the WPDI group to report more favorable experiences due to perceived additional attention. Since patient experience is a subjective primary outcome, these results may partially reflect this awareness rather than the intervention's efficacy. Consequently, findings should be interpreted with caution. Future research employing blinded assessments or objective measures is necessary to mitigate this potential bias and further validate the observed improvements. Third, although physicians encouraged all patients in the WPDI group to participate in the WeChat interaction, some participants remained silent throughout the treatment period. However, our review of the group discussions suggested that these patients either had no concerns about the treatment or found that their questions had already been addressed by others. Finally, despite efforts by physicians to respond promptly to all questions in the group chat, occasional delays in responses still occurred. Nevertheless, most responses were provided within one hour, and patient experience and compliance in the WPDI group did not appear to be substantially affected. In the future, the integration of artificial intelligence-assisted systems may help provide more timely responses and further improve communication efficiency.

## 5 Conclusion

The implementation of WPDI did improve the patients' treatment experience significantly. However, it did not improve the treatment outcomes of *H. pylori* eradication therapy. So far, adequate routine education, including oral and written instruction, is important and remains necessary for *H. pylori* eradication. In the future, physicians should focus on not only improving the eradication rate of *H. pylori* therapy but also promoting the patient experience, and artificial intelligence may serve as an assistant tool.

## Data Availability

The raw data supporting the conclusions of this article will be made available by the authors, without undue reservation.
